# Tailoring the Evidence for Exercise Oncology within Breast Cancer Care

**DOI:** 10.3390/curroncol29070383

**Published:** 2022-07-09

**Authors:** Chad W. Wagoner, Lauren C. Capozzi, S. Nicole Culos-Reed

**Affiliations:** 1 Faculty of Kinesiology, University of Calgary, Calgary, AB T2N 1N4, Canada; chad.wagoner@ucalgary.ca (C.W.W.); lcapozzi@ucalgary.ca (L.C.C.); 2 Department of Clinical Neurosciences, Cumming School of Medicine, University of Calgary, Calgary, AB T2N 1N4, Canada; 3 Department of Oncology, Cumming School of Medicine, University of Calgary, Calgary, AB T2N 1N4, Canada; 4 Department of Psychosocial Resources, Tom Baker Cancer Centre, Cancer Care, Alberta Health Services, Calgary, AB T2N 4N2, Canada

**Keywords:** breast cancer, oncology, exercise tailoring, standard cancer care, prehabilitation, rehabilitation, physical activity, implementation, physical function

## Abstract

Exercise is safe and effective for those living with and beyond breast cancer, with evidence supporting exercise guidelines, and position statements from international organizations. Despite the clearly recognized benefits of exercise for these individuals, many do not participate or maintain recommended exercise levels throughout the breast cancer continuum, highlighting the lack of translation from research into practice. In addition, discerning how exercise can be tailored to address breast cancer-related impairments, so that individuals are able to participate safely and effectively, has also not been studied extensively. Thus, we propose that implementing exercise screening, triage, and referral pathways across the breast cancer continuum may allow for increased accessibility and adoption among those living with and beyond breast cancer. This paper provides an overview of exercise prescription tailoring for common breast cancer and treatment-related impairments, proposes a simplified screening tool for identifying physical activity and movement-related impairments, and considers how best to channel evidence into practice via proposed implementation pathways that may better connect individuals living with and beyond breast cancer with exercise oncology resources through screening, triage, and referral.

## 1. Background

The impact of exercise has been consistently studied within distinct phases of the breast cancer continuum, including pre-breast-cancer treatment (i.e., prehabilitation), during-breast-cancer treatment, after-breast-cancer treatment into survivorship, and living with metastatic breast cancer. In each of these phases, evidence has shown that exercise participation is a safe, viable, and effective means of providing functional, fitness, psychosocial, and treatment-related benefits [1,2,3,4]. This has resulted in exercise guidelines and recommendations for all individuals living with and beyond cancer, further supporting the need to embed exercise into clinical care pathways. Both the British Association of Sport and Exercise Sciences (BASES) and Exercise and Sport Science Australia (ESSA) have published position statements for including exercise as a part of cancer care, in addition to calling for individualized exercise prescriptions and incorporating behavior change support [5,6]. Similarly, the American College of Sports Medicine (ACSM) has convened two roundtables since 2010 to publish cancer-specific exercise guidelines, with the most recent 2019 guidelines including evidence addressing common cancer-related side effects, and strategies for engaging clinicians [7,8,9].

Although the safety and benefits of exercise for those living with and beyond breast cancer have been established, reports continue to show that overall physical activity levels amongst the breast cancer population are low across the continuum [10,11]. A recent study identified both physical (i.e., fatigue) and psychosocial (i.e., lack of motivation) outcomes as being barriers to exercise participation within the breast cancer population [12]. This highlights the need for a tailored exercise prescription approach to address cancer-related impairments (i.e., functional, fitness, or psychosocial decline) that may occur across multiple phases of the breast cancer continuum, as well as behavior-change support to promote self-efficacy, establish exercise-related goals, and maintain exercise participation long-term. Additionally, exercise is not yet considered part of standard supportive cancer care—not even in breast cancer, where the bulk of the evidence exists [13,14]. Barriers continue to persist for healthcare providers to refer to exercise, and for those with breast cancer to access exercise resources and programs [15,16]. To address this, a culture shift in cancer care must occur, with clinical care pathways developed and implemented to screen, identify, and triage individuals for appropriate exercise and rehabilitation services throughout the breast cancer continuum. This paper provides an overview of exercise-tailoring recommendations to address breast-cancer-specific impairments, discusses the importance of behavior-change support within exercise oncology, and addresses how to better channel evidence into practice via proposed screening, triage, and referral pathways for embedding exercise within breast cancer care.

## 2. Exercise Prescription to Address Cancer-Related Impairments: A Tailored Approach

In addition to consideration of whether a patient is pre-, during-, or post-treatment, exercise prescription tailoring is dependent on individual cancer-related and other disease-related or functional impairments. Individuals living with breast cancer often face a myriad of functional, psychosocial, fitness, and symptom-related impairments that impact their exercise tolerance, goals, and response to an exercise intervention [17,18]. Some living with breast cancer may also have pre-morbid or comorbid conditions that require exercise tailoring (i.e., previous musculoskeletal injuries, heart disease, diabetes) [19]. Thus, screening for both breast-cancer-specific and other disease or functional impairments is required, to safely and effectively tailor exercise prescriptions and recommendations within any phase of the breast cancer continuum. This section will review common breast-cancer-related impairments and provide exercise-tailoring recommendations based on the current state of the literature. It should be noted that more research is required for screening and tailoring for specific impairments within exercise oncology.

### 2.1. Functional Impairment

#### 2.1.1. Chemotherapy-Induced Peripheral Neuropathy (CIPN)

CIPN is a polyneuropathy that usually presents with numbness and tingling in a glove and stocking pattern, and is associated with difficulties with upper and lower extremity function, gait ataxia, and decreased quality of life (QOL) [20]. It affects up to 68% of patients treated with neurotoxic chemotherapies, and can have lasting effects in up to 40% of individuals [20]. Exercise interventions in patients receiving neurotoxic chemotherapies [20], and after treatment completion [21], have examined the impact on CIPN symptoms and neurophysiology, physical function, disability, and QOL. During treatment, a combined walking and resistance training intervention was found to reduce CIPN paresthesia symptoms in the hands and feet, when compared to the non-exercise group [21]. McCray and colleagues, investigating the impact of exercise following treatment, found that an 8-week aerobic, resistance, and balance training intervention led to significant improvements in CIPN symptom management, dynamic and standing balance, mobility, and QOL when compared to control [20].

##### Tailoring Considerations

Individuals at risk for, or living with, CIPN should, as tolerated, aim to meet the current ACSM Physical Activity guidelines of 90 min of moderate-to-vigorous aerobic exercise per week, plus at least 2 days of resistance exercise, and flexibility training most days of the week [7]. In accordance with the guidelines, if symptoms impact grip, then bands should be used or bodyweight exercises, rather than heavy weights, which may be more difficult to hold, and may cause injury if dropped. If gait ataxia is present, then seated or closed-chain exercise options should be chosen, to limit falls. Further medical consultation will be necessary if there is a progression or change in neuropathy symptoms.

#### 2.1.2. Lymphedema

Lymphedema is an accumulation of protein-rich lymphatic fluid due to ineffective lymphatic drainage, leading to regional swelling [22]. In breast cancer, it presents as swelling of the arm on the affected side. It is most common in individuals who have had surgical resection of lymph tissue, or radiation affecting the lymphatic system [22]. Most patients present with pain, heaviness, or tightness in the affected arm, decreased range of motion at the fingers, wrist or elbow joints, decreased functioning of the hand and arm, and decreased QOL [22]. It is a manageable but not curable condition, which affects up to 20% of breast cancer patients [22]. Physiotherapists and Certified Lymphedema Therapists are often involved in the care and management of individuals with lymphedema, targeting lymph drainage and the implementation of tools like compression garments. In the early 2000s, evidence emerged showing a lack of associated exacerbation of lymphedema with structured exercise [23]. Since then, more evidence from studying the role of mainly resistance exercise has shown that it is effective in increasing fitness and muscle mass without exacerbating fluid volume in the affected limb [22]. There has been less work on examining the role of aerobic exercise on lymphedema symptom management, but previous studies indicate that it is not associated with any worsening of lymphedema symptoms [24].

##### Tailoring Considerations

Exercise plays an important role in lymphedema symptom management and preservation of upper extremity limb function. Lymphedema should be controlled prior to starting a new exercise program and should be monitored during and after exercise sessions. Exercise should be at least partially supervised by a qualified exercise professional (QEP), and a focus on slow and progressive resistance exercise, at a light-to-moderate intensity, is recommended. Compression garments should be worn as prescribed.

#### 2.1.3. Post-Mastectomy Pain Syndrome

Post-Mastectomy Pain Syndrome (PMPS) is a neuropathic pain condition affecting upwards of 25–60% of individuals living with breast cancer after surgical treatment [25]. Individuals present with burning or numbness around the breast, chest wall, axilla, and medial arm on the affected side, and it is associated with damage to the nervous tissue in the intercostobrachial, medial pectoral, lateral pectoral, and thoracodorsal or long thoracic nerves [25]. Traditional therapies include focal or systemic neuropathic pain treatments, but more recent evidence points to the role of exercise in decreasing pain severity and improving QOL among women affected by PMPS [25]. Specifically, a systematic review evaluating aerobic, resistance, stretching, and aqua fitness interventions showed improved general, physical, and mental QOL scores, and reduction in pain scores between the exercise versus control groups [25].

##### Tailoring Considerations

Individuals with PMPS may be living with ongoing pain that may impact their exercise participation: it is important to educate them in the awareness that regular exercise participation can help to manage pain symptoms, and that exercises can be modified so that the pain symptoms do not worsen. A feasible and informative strategy to tailor exercise for pain management is to implement a visual analog pain scale before and after exercise sessions, which can provide helpful feedback when considering program modifications. Exercise movements should not further exacerbate symptoms or be a source of new pain.

#### 2.1.4. Osteoporosis

Osteoporosis is a progressive disease characterized by a decrease in bone mineral density, leading to an increased risk of bone fragility and fracture [26]. Breast cancer treatments, including many chemotherapeutic agents and aromatase inhibitors, increase the risk of osteoporosis, as do many common comorbidities such as increasing age, genetic factors, lifestyle factors, and endocrine disorders. Exercise for breast cancer patients improves bone density and prevents the risk of fractures [27,28,29].

##### Tailoring Considerations

Focus on bone-loading activities to improve bone integrity and density. Some of these activities may include hiking, walking, climbing stairs, weightlifting, and performing body weight exercises. Increase regular weight-bearing exercises and muscle strengthening exercises to load bone and improve balance, as well as preventing falls. To reduce the risk of falls, ensure a safe environment for exercise with no loose rugs or mats on the floor, removal of any obstacles, use of well-fitting shoes and good room lighting. When necessary, provide supports for balance, to prevent falls or for modification of movement, to increase balance awareness.

#### 2.1.5. Bone Metastasis

Upwards of 70% of breast cancer patients with metastatic disease experience bone metastasis [29]. Patients may present with debilitating pain, pathologic fractures, and spinal cord compression as a consequence of boney metastasis [30]. Due to concerns regarding pathologic fractures, in the past, limited rehabilitation and exercise interventions were implemented for this population [31]. Newer evidence shows that exercise is safe, feasible, and beneficial for people living with bone metastasis, and although much more research is needed, early work in mixed metastatic cancer groups shows that exercise is important in preventing deconditioning and improving QOL [32].

##### Tailoring Considerations

It is important to obtain a physician consultation prior to starting a new exercise program for someone with bone metastasis. Understanding the extent of the boney metastasis allows for tailoring of exercise with the goal of improving overall conditioning, while avoiding excessive torque across the affected metastatic bone. Unlike osteoporotic bone, bone-loading does not increase bone integrity for those with boney metastasis [33]. Avoid any activities that cause bone pain and avoid resisted flexion and extension at the hip if metastatic disease is to the pelvis or femur. Limit simultaneous bending and lifting or twisting and lifting if there is metastatic disease to the axial skeleton [33]. Instead, choose partial-weight-bearing activities, or assisted movements such as pull-ups, push-ups, or dips, if the patient does not have upper extremity metastasis.

### 2.2. Exercise Tolerance and Body Composition

#### 2.2.1. Cardiotoxicities

Breast cancer treatment may involve cardiotoxic therapies such as anthracyclines, alkylating agents, and monoclonal antibody-based tyrosine kinase inhibitors, which can significantly impair left ventricular ejection fraction by more than 10%, to a value less than 53% [34]. These individuals may experience chest pain, palpitations, changes in blood pressure, or light-headedness. Although exercise for individuals with cardiovascular disease has been well-established for decades, exercise specifically for those with cardiotoxic effects due to cancer treatment is less studied [35]. Early work suggests that aerobic exercise may play a key role in prevention of cardiac changes during treatment and promotion of cardiovascular health following these treatments [36,37].

##### Tailoring Consideration

Individuals with breast cancer who are on or have received cardiotoxic treatments should have a consultation with a physician prior to starting or changing their exercise routine. They may undergo cardiac testing and receive specific heart rate ranges for exercise. Monitoring exercise intensity using the Borg’s Rating of Perceived Exertion (RPE; see Table 1) scale [38] is also useful to ensure an appropriate response to exercise. Start with low-to-moderate aerobic exercise to build fitness and functional ability, and progress slowly over time with tolerance.

#### 2.2.2. Body Composition and Weight Management

At diagnoses, it is estimated that approximately 65% of breast cancer patients are overweight or obese, with a body mass index (BMI) of greater than 25 kg/m^2^ [28]. Obesity after diagnosis is linked to poorer outcomes, including increased risk of cancer recurrence and mortality [39]. Furthermore, breast cancer treatments are associated with increased body fat as well as decreased lean body mass, which can impact overall function and QOL, and decrease response and adherence to aromatase inhibitors [40,41,42]. Therefore, interventions that help to manage weight and body composition are essential. Exercise interventions targeting body composition in breast cancer patients decrease percent body fat and BMI while increasing lean body mass [28]. Specifically, Thomas and colleagues investigated a year-long intervention, with the exercise intervention group receiving twice-weekly resistance sessions and 150 minutes of aerobic exercise. The results indicated that the exercise intervention group had an increase of 0.32 kg in lean body mass versus −0.88 kg in the controls, and a decrease of −1.4% of body fat compared to −0.48% in the control group [28].

##### Tailoring Considerations

A multimodal exercise program may provide optimal body-composition and weight-management benefits [43]. Progressive resistance exercise, with sessions at least two times per week, can preserve and restore muscle mass. Cardiovascular training, focusing on endurance and interval-type exercise, can be useful for weight loss specifically [43]. Referral to a dietitian can further help patients with healthy eating habits to facilitate weight loss or weight maintenance. In addition, behavior-change support—including health coaching with goal setting, action planning, and strategies to combat barriers—is an important consideration for an effective weight-management program.

### 2.3. Psychosocial Well-Being

#### 2.3.1. Stress, Anxiety, Depression, and Mood

Anxiety, depression, and overall distress are commonly reported symptoms experienced by those living with breast cancer, from diagnosis onwards [44]. Rates of distress can vary across the cancer continuum, but may require an interdisciplinary team including oncologists, family physicians, psychologists, psychiatrists, social workers, and other healthcare professionals (such as palliative care physicians) to assist patients in the management of their moods, and with coping, well-being, and overall QOL. Exercise and other movement therapies, like yoga and Pilates, improve rates of depression and anxiety among women with breast cancer [45]. For example, patients in an 8-week Pilates intervention had significant improvements on the Beck Depression Index when compared to the control group [46], while a recent systematic examination of 12 aerobic exercise studies found alleviation of depression and anxiety [47]. Across the exercise and mental health literature in general, and specific to cancer, there is no ‘ideal’ dose in terms of FITT exercise prescription. However, slightly longer duration (upwards of 10+ weeks) and more frequent weekly sessions (2 or more days per week of exercise) appears to optimize well-being, reduce indices of emotional distress, and enhance overall QOL [7].

##### Tailoring Consideration

Educate patients on the evidence supporting the role of exercise in the management of stress, anxiety, and depression, as well as its effect on overall mood, well-being, and QOL (Resources—Table 1). Patients can use visual analog scales to rate feelings (depression, anxiety, stress, mood in general) before and after an exercise session, to track the impact of exercise on their well-being. With the experience of exercise’s positive impact on these indices of emotional distress, participants may be more likely to utilize exercise as a trusted resource to enhance their mental health. In addition, facilitating social support, a positive motivational climate, and providing education on exercise-behavior-change strategies and resources, may lead to better adoption and adherence to exercise [7]. Specifically, to effectively tailor exercise for mental health benefits, asking participants about their exercise preferences, providing choice in activities (to enhance their sense of control), building a positive motivational climate in the exercise setting, utilizing social connections and social support, and supporting participant development of key behavior-change skills (goal setting, barrier management, planning intentions) will support exercise adherence and positively impact well-being. Resources to support QEPs to develop and utilize these skills within exercise settings (general and specific to oncology) are available from numerous training sources (see Table 1).

### 2.4. Symptom-Related Impairment

#### 2.4.1. Cancer-Related Fatigue

Studies have reported upwards of 99% of breast cancer patients report chronic cancer-related fatigue (CRF) following their diagnosis, impacting QOL, function, and return to work [48,49]. Both aerobic and resistance exercise play a key role in decreasing and managing CRF, with a recent systematic review showing increased benefit among programs with longer length, duration, and frequency of the supervised sessions [50,51,52].

##### Tailoring Considerations

Exercise is a key component in the management of CRF, and inactivity should be avoided. It is important to educate those with breast cancer on the role of exercise, as this can help to eliminate barriers and misinformation. Use a visual analogue scale (VAS) to measure fatigue before and after an exercise session, and aim for either the same or lower fatigue levels post-exercise [33]. In addition, measuring ‘energy’ levels with a VAS or a ‘thermometer’ style tool can be beneficial for ensuring that, both during and after an exercise session, energy is gained while fatigue is not enhanced (and may be improved). Individuals should not feel that exercise worsens their fatigue. On days when fatigue is higher, choose lower-intensity exercise, easier modifications, or gentler movement options.

## 3. Behavior-Change Support within Breast Cancer Care

In addition to tailoring exercise prescriptions, behavior-change support at individual (i.e., goal setting, barrier management, action planning, social support) and systems levels (i.e., administrative support for exercise resources, role of the health care providers, access to educational and program resources) is critical for facilitating engagement in exercise, and promotion of increased overall physical activity levels across the breast cancer continuum. Within our work, we implement an ‘Exercise and Educate’ approach [53,54,55,56], where patients are provided educational material on exercise benefits pre-, during-, or post-treatment, and specific behavior-change techniques (i.e., goal setting, action planning, self-monitoring) within the evidence-based exercise oncology program. Importantly, this behavior-change education is also included within the training pathway for the QEPs leading the exercise oncology program (https://thrivehealthservices.com accessed on 31 March 2022). With this behavior-change training, the QEP can then embed the support into a positive motivational climate within each class (i.e., Exercise and Educate), supporting the participant with handouts on each topic and guided discussions during class (warm-up and cool down), as well as brief post-class discussions. This Exercise and Educate approach thus builds the skills needed for successful behavior change, beyond simply providing the exercise prescription, promoting sustainable behavior change for exercise adoption, adherence, and maintenance.

This Exercise and Educate approach for exercise professionals and participants is then delivered within a larger ‘clinic-to-community’ model, designed to increase accessibility to exercise oncology programs. As one example, in Canada, ongoing work is examining a nationally delivered exercise program which includes the Exercise and Educate approach in a clinic-to-community model (NCT04478851). The EXercise for Cancer to Enhance Living Well (EXCEL) study offers online delivery of an exercise oncology 12-week program to individuals living with cancer that have limited access to exercise oncology resources, with a specific focus on rural/remote populations. In addition to improving accessibility to more individuals living with cancer outside of urban areas, EXCEL implements the Exercise and Educate approach with professional fitness training, handouts to participants, and further behavior-change webinars to provide participants with the tools necessary to continue to remain physically active after the exercise intervention. A summary of education topics and targeted behavior-change techniques that occur throughout the EXCEL 12-week exercise intervention is provided in Table 2.

This behavior-change support enhances participant engagement, fosters social support between participants and the fitness professionals, and builds the skills and resources that are necessary to foster physical activity adherence and maintenance. EXCEL also employs the clinic-to-community model, with clinical exercise physiologists (CEP) and medical expertise at central (urban) ‘hubs’ to facilitate the development of both healthcare provider (HCP) and fitness professional networks at ‘spokes’ across rural and remote locations. These networks facilitate referral and exercise oncology program delivery (via QEPs) for sustainable implementation of the EXCEL intervention. Additionally, our multiphasic prehabilitation intervention for those living with head and neck cancer (NCT04598087), the Exercise and Educate approach is provided across the entirety of the treatment continuum (pre-surgery, peri-operatively, post-surgery, and recovery phases). This includes movement goals tailored for each phase: a functional-movement focus pre-surgery, an early-mobility focus peri-operatively, and structured exercise prescription post-surgery and into recovery. Participants are provided with an educational workbook to support their exercise-behavior change, along with support from a CEP with health coaching expertise.

Importantly, the Exercise and Educate model is adaptable, as it is recognized that there are different strategies and techniques that can be provided to support physical activity and exercise-behavior change. While the evidence supports behavior-change skills as critical for building and maintaining exercise, this evidence is inconclusive in terms of which specific techniques and applied theories (i.e., Theory of Planned Behavior) may support optimal physical activity and exercise engagement [57]. Thus, interventions utilizing an Exercise and Educate approach should be flexible and tailored to address specific tumor group needs for each individual (i.e., lymphedema, body composition, mental wellness, or other symptom management needs in breast cancer). This approach may provide those with breast cancer the best chance possible to improve or maintain physical function, build exercise behaviors, and maintain or enhance overall QOL across the breast cancer continuum. To do this effectively, adapting cancer care for the supportive inclusion of exercise resources for those living with breast cancer is essential. Breast cancer patients and survivors must be able to access the right resource at the right time, to safely and adequately address specific needs.

## 4. Exercise as Part of Standard Breast Cancer Care

**Figure 1:**
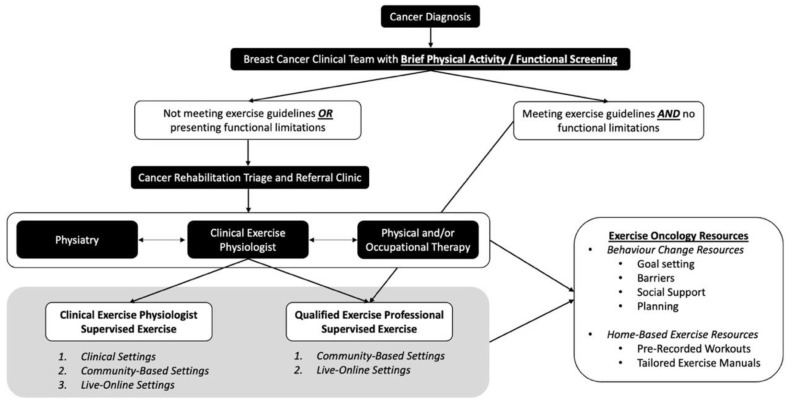
Breast Cancer Pathway for Integration of Exercise Oncology: Screening, Triage, and Referral.

Given the amount of established exercise and breast cancer evidence, and translation of this evidence into exercise guidelines and recommendations [5,6,7], we must consider how to sustainably implement exercise into standard breast cancer supportive care. Previous work has emphasized the importance and complexity of exercise screening, triage, and referral within clinical cancer care, proposing potential algorithms (e.g., EXCEEDS algorithm) to direct patients to the appropriate exercise resource [58,59]. In our original pathways model [60], we have a CEP as the critical link between the HCPs and the required exercise screening and resources that are provided in a tailored exercise approach for each individual. CEPs have a degree in Kinesiology or Exercise Science, and extra post-graduate training and certification to work specifically with clinical populations. Considering breast-cancer-specific needs across pre-treatment, treatment, post-treatment, and while living with metastatic breast cancer, we have proposed an updated “Breast Cancer Pathways” model (See Figure 1). This model includes further screening for functional impairment [61], the role of cancer specialists in rehabilitation medicine, and the role of CEPs within clinical care and QEPs (including group fitness instructors, personal trainers, and other trained fitness designations) within the community. A transdisciplinary team approach, and developing partnerships within clinical and community settings, will ultimately support the safe, effective, and sustainable implementation of exercise as an evidence-based supportive cancer care resource to enhance health, wellness, and overall QOL in individuals living with and beyond breast cancer.

In this “Breast Cancer Pathways” model, we propose a simplified screening system to identify the tailored exercise resources that will meet the functional, physical activity, and exercise needs of individuals living with and beyond breast cancer (top tier of Figure 1). This includes implementation of a Brief Physical Activity/Functional Screening Questionnaire (Table 3) that can be easily administered by the HCPs (i.e., nurse, oncologist). This screening is designed to be simple and short, allowing it to be feasibly implemented within a busy clinical setting, while also directing patients to the proper cancer rehabilitation and exercise resources. Specifically, if not physically active or if functional limitations/comorbidities are identified, HCPs can refer for further screening to a Cancer Rehabilitation Triage and Referral clinic (middle tier of Figure 1), where patients are further assessed, and then provided with the appropriate exercise and/or rehabilitation referrals (i.e., Physiatry, Physical or Occupational Therapy, or CEP for exercise resource access).

This concept is currently being implemented within a neuro-oncology clinic (ACE-Neuro: NCT04831190), to better understand pathway feasibility. In this Cancer Rehabilitation Triage and Referral Clinic, a complete medical and cancer-specific history is collected, and a neurologic and musculoskeletal examination is performed. Functional performance batteries like the Short Physical Performance Battery can also be performed, to better assess physical function [62]. From here, a Karnofsky Performance Scale score [63] or ECOG (Eastern Cooperative Oncology Group) [64] score is determined, and specific physical, cognitive or psychosocial impairments are identified.

Considering its application to breast cancer, the triage and referral clinic provides the ability to identify and address breast-cancer-related impairments with the appropriate resources. If issues such as pain syndromes, bone metastasis, uncontrolled lymphedema, CIPN, or functional concerns impacting the activities of daily living are identified, then patients can be referred for further assessment and development of a tailored plan by a cancer physiatrist. For those with functional impairment, including shoulder dysfunction, lymphedema, return-to-work-related issues, or mobility issues, a referral to physical therapy and/or occupational therapy is warranted. Those who are stable from a medical and neurological perspective, but have ongoing symptom management or well-being concerns, or who are not currently meeting the exercise guidelines, are referred to a CEP. Importantly, decisions may result in a referral to the multiple supportive cancer care resources described above, to address simultaneous functional and physical activity limitations, and to support attaining exercise guidelines levels, ensuring that the right resource is referred at the right time, tailored to the individual’s needs.

The bottom tier of Figure 1 focuses on physical activity promotion and exercise program implementation via supervised exercise with a CEP, or work with a QEP in the community. For those who are not currently physically active, have identified functional deficits or comorbidities (i.e., treatment-related cardiotoxic impairments), or who have a metastatic cancer diagnosis, a referral to a CEP within the clinical or community setting is most appropriate. A CEP with exercise oncology training (Training Resources—Table 1) has the highest level of training in the exercise oncology field. They have the scope of practice to provide further exercise-specific screening, to tailor exercise prescription based on patient needs, and to supervise exercise sessions for those deemed high-risk within clinical and community-based settings; they should also have the training to provide the necessary exercise-behavior-change support (i.e., health coaching for communication approaches, integration of behavior-change skills). In addition, the CEP can facilitate referral to evidenced-based exercise oncology resources delivered by a trained QEP within the community, when and if a participant is deemed eligible for that level of supervision. Individuals who are meeting exercise guidelines, and who do not have functional limitations or comorbidities, may be referred to the QEP and provided with exercise oncology resources within the community. QEPs include those with a recognized exercise delivery certification (e.g., certified personal trainers or group fitness instructors), with exercise oncology training. In this setting, QEPs can also provide patients with exercise oncology resources for exercising on their own. It should be noted that this pathway is not linear, as functional and physical activity setbacks happen. In this instance, QEPs and CEPs must have a communication pathway back to the other cancer care supportive resources (Physiatry, Physical/Occupational Therapy) for delivery of the appropriate supportive cancer care resource. These open lines of communication are essential for sustainable exercise referral pathways and will ensure that individuals living with and beyond breast cancer receive the appropriate care.

In channeling exercise evidence into practice, it is critical to consider trial designs that will support understanding of intervention effectiveness, the ability to successfully deliver exercise resources (education and programming), and sustainable exercise behavior change (i.e., a more active population of individuals living with and beyond breast cancer). Sequential Multiple Assignment Randomized Trials (SMARTs) are viable research designs that aim to develop and evaluate adaptive interventions that are both feasible and impactful in practice [65]. Specifically, SMARTs have been implemented to improve physical activity levels and weight loss [66,67], and may be suitable to implement, assess, and adapt exercise interventions within clinical cancer care settings, as they allow for tailoring at various intervention timepoints.

Furthermore, if HCP barriers to exercise referral—such as lack of time, knowledge, and exercise resources—are not adequately addressed [16], exercise engagement will continue to remain low across the breast cancer continuum. To address this, HCPs must have the awareness and ability to refer to a CEP within the clinical setting, to facilitate screening, triage, and referral, so that patients can access clinic or community-based exercise resources. Referral must be simple for HCPs, to accommodate busy clinical settings, and having the simple step of a triage clinic, that is supported by a CEP within the clinical setting, can ensure that this occurs seamlessly for all HCPs, and thus improves the embedding of exercise into supportive cancer care.

Finally, the ongoing development of educational resources for QEPs, and efforts to build community partnerships with these exercise professionals, will improve community capacity, thereby enhancing access to community-based exercise programs for individuals living with and beyond cancer, and supporting sustainable exercise-behavior change. Without this, many individuals will continue to be unable to experience exercise benefits, including managing treatment-related side-effects and optimizing physical and psychosocial outcomes. Together, implementation and evaluation of sustainable “clinic-to-community” screening, triage, and referral pathways to connect individuals living with breast cancer to exercise oncology resources, will ensure that exercise becomes an integral part of standard breast cancer supportive care. Future implementation efforts are thus critical for identifying how to make exercise participation across the entirety of the breast cancer continuum feasible, sustainable, and effective.

## 5. Summary

The evidence clearly supports the benefits of exercise across the breast cancer continuum. There are unique needs that can be addressed (i.e., symptom management, enhanced QOL, emotional well-being, and challenges with behavior change), with tailoring of exercise to best suit the needs of those living with and beyond breast cancer. This article reviews initial suggestions for tailoring, but many gaps remain, and research is necessary, to better refine exercise prescription to optimize benefits—physical and psychosocial. To maximize the adoption, adherence, and maintenance of exercise, behavior-change strategies must be included in physical activity programming. Our Exercise and Educate approach at the program delivery level, with training for QEPs, and at the program level, with resources for participants, is essential for supporting exercise adherence and longer-term maintenance.

Finally, the logistics of how breast cancer survivors are screened, triaged, and referred to exercise oncology programs, is key for supporting wellness via exercise resources in this population. A clinic-to-community framework, that builds networks between oncology clinics and community exercise resources, will best support the moving of patients from the clinical setting to appropriate community-based exercise programs. This clinic-to-community model ultimately requires building a link between these two settings. We propose that this is best done via the integration of CEPs into cancer care, and the adoption of the Breast Cancer Pathway for Integration of Exercise Oncology model, which outlines a simplified screening, triage, and referral approach, ensuring that those living with and beyond breast cancer are identified and referred to the appropriate transdisciplinary HCP teams (e.g., physiatry, rehabilitation, exercise). Ultimately, this will result in the right exercise resource being tailored to meet unique needs at the right time, enabling more individuals living with and beyond breast cancer to adopt, and benefit from, physical activity and an active lifestyle.

## Figures and Tables

**Table 1:** Qualified Exercise Professional Resources for Exercise Oncology Delivery *.

**Exercise Oncology Specific Certifications/Trainings**
▪ *American College of Sports Medicine (ACSM) Cancer Exercise Trainer* o https://www.acsm.org/certification/specialized ▪ *CanRehab* o https://www.canrehab.com ▪ Thrive Health Services Cancer and Exercise Training o https://thrivehealthservices.com ▪ *Ex-Med Cancer* o https://www.exmedcancer.org.au ▪ *Exercise Medicine Research Institute* o https://www.exercisemedicine.org.au/professional-development/exercise-oncology
**Exercise and Mental Health Resources**
▪ *The Centre for Addiction and Mental Health (CAMH)* o https://www.camh.ca ▪ *Canadian Society of Exercise Physiology (CSEP)—Mental Health and Exercise* o https://store.csep.ca/products/advanced-learning-module-mental-health-and-exercise
**Exercise Delivery Resources**
▪ *Rate of Perceived Exertion Scale* o https://thrivehealthservices.com/RPEScale.pdf ▪ *Behavior-Change Stages and Strategies* o https://thrivehealthservices.com/BehaviourChangeStages.pdf o https://thrivehealthservices.com/BehaviourChangeStagesStrategies.pdf o https://thrivehealthservices.com/BehaviourChangeStrategies.pdf

* All online resources accessed on 31 March 2022.

**Table 2:** “Exercise and Educate” Summary Table.

Education Topics	Areas Covered	Behavior-Change Technique
*Principles of Exercise and Cancer*	Physical and psychosocial benefits of exercise Exercise principles Exercise progression (i.e., FITT Principle) Safety during exercise	Education Support
*Physical Activity Tracking*	Benefits of self-monitoring for physical activity Different forms of activity tracking o Activity Trackers, Smartphone Applications, Exercise Logs	Self-Monitoring
*Goal Setting*	SMAR^2^T^2^ goal setting Process vs. outcome goals	Goal Setting
*Behavior Change and Relapse Prevention*	Identifying barriers Planning for barriers Positive behavior change Positive motivational climate Maintaining motivation	Barrier Management Communication Skills
*Fatigue and Stress Management*	Common definitions of stress and fatigue Fatigue and stress relationship with exercise and physical activity Relaxation techniques for stress management	Barrier Management
*Social Support and Long-Term Maintenance*	Identifying sources of social support Tips for maintaining exercise and physical activity	Social Support

**Table 3:** Brief Physical Activity/Functional Screening Questionnaire.

Physical Activity Screening *	
*(1) To the best of your knowledge, do you currently meet any of the following exercise guidelines? Select ‘Yes’ if any one of these apply:* 90 min/week of moderate aerobic exercise (e.g., light jogging for bouts ≥ 10 min) 75 min/week of vigorous aerobic exercise (e.g., running for bouts ≥ 10 min) 2 days/week of strength training that works all major muscle groups ***in addition to*** any of the above aerobic exercise recommendations listed above	Yes/No
**Movement/Functional Screening ^†^**	
*(2) Are you currently experiencing any of the following side-effects from the breast cancer diagnosis and/or associated treatment? Select ‘Yes’ if any one of these apply:* Swelling in your arms or hands (Lymphedema) Loss of range of motion at your shoulder(s) Numbness, tingling or pain in your hands or feet (Peripheral Neuropathy) Pain in the chest wall or armpit Shortness of breath Significant changes in body weight Debilitating fatigue Pain that limits your ability to carry out activities of daily living Increased risk of bone fracture (i.e., osteoporosis or bone metastases)	Yes/No
*(3) Have you been told to manage, or are you taking, any medication for any of the following conditions? Select ‘Yes’ if any one of these apply:* Heart Diseases High Blood Pressure Respiratory Diseases Diabetes Weight Management Arthritis	Yes/No

* Answering ‘No’ to physical activity screening question triggers referral to triage clinic ^†^ Answering ‘Yes’ to either movement/functional screening question triggers referral to triage clinic.

## Data Availability

Not applicable.

## References

[1] Cannioto R.A., Hutson A., Dighe S., McCann W., McCann S.E., Zirpoli G.R., Barlow W., Kelly K.M., DeNysschen C.A., Hershman D.L. (2021). Physical Activity Before, During, and After Chemotherapy for High-Risk Breast Cancer: Relationships With Survival. JNCI J. Natl. Cancer Inst..

[2] Schmid D., Leitzmann M.F. (2014). Association between physical activity and mortality among breast cancer and colorectal cancer survivors: A systematic review and meta-analysis. Ann. Oncol..

[3] Battaglini C.L., Mills R.C., Phillips B.L., Lee J.T., Story C.E., Nascimento M.G., Hackney A.C. (2014). Twenty-five years of research on the effects of exercise training in breast cancer survivors: A systematic review of the literature. World J. Clin. Oncol..

[4] Yang L., Morielli A.R., Heer E., Kirkham A.A., Cheung W.Y., Usmani N., Friedenreich C.M., Courneya K.S. (2021). Effects of Exercise on Cancer Treatment Efficacy: A Systematic Review of Preclinical and Clinical Studies. Cancer Res..

[5] Hayes S.C., Newton R.U., Spence R.R., Galvão D.A. (2019). The Exercise and Sports Science Australia position statement: Exercise medicine in cancer management. J. Sci. Med. Sport.

[6] Campbell A., Stevinson C., Crank H. (2012). The BASES Expert Statement on Exercise and Cancer Survivorship. J. Sports Sci..

[7] Campbell K.L., Winters-Stone K.M., Wiskemann J., May A.M., Schwartz A.L., Courneya K.S., Zucker D.S., Matthews C.E., Ligibel J.A., Gerber L.H. (2019). Exercise Guidelines for Cancer Survivors: Consensus Statement from International Multidisciplinary Roundtable. Med. Sci. Sports Exerc..

[8] Schmitz K.H., Campbell A.M., Stuiver M.M., Pinto B.M., Schwartz A.L., Morris G.S., Ligibel J.A., Cheville A., Galvão D.A., Alfano C.M. (2019). Exercise is medicine in oncology: Engaging clinicians to help patients move through cancer. CA Cancer J. Clin..

[9] Patel A.V., Friedenreich C.M., Moore S.C., Hayes S.C., Silver J.K., Campbell K.L., Winters-Stone K., Gerber L.H., George S.M., Fulton J.E. (2019). American College of Sports Medicine Roundtable Report on Physical Activity, Sedentary Behavior, and Cancer Prevention and Control. Med. Sci. Sports Exerc..

[10] De Groef A., Geraerts I., Demeyer H., Van der Gucht E., Dams L., de Kinkelder C., Dukers-van Althuis S., Van Kampen M., Devoogdt N. (2018). Physical activity levels after treatment for breast cancer: Two-year follow-up. Breast.

[11] De Groef A., Demeyer H., de Kinkelder C., Dukers-van Althuis S., Asnong A., Dams L., Van der Gucht E., De Vrieze T., Haenen V., Evenepoel M. (2022). Physical Activity Levels of Breast Cancer Patients Before Diagnosis Compared to a Reference Population: A Cross-Sectional Comparative Study. Clin. Breast Cancer.

[12] Schneider C., Reimann S., Schmid J., Bernhard J., Rabaglio M., Campbell K.L., Wilhelm M., Eser P. (2022). Qualitative analysis of facilitators and barriers to centre- and home-based exercise training in breast cancer patients—A Swiss tertiary centre experience. Swiss Med. Wkly..

[13] Lahart I.M., Metsios G.S., Nevill A.M., Carmichael A.R. (2018). Physical activity for women with breast cancer after adjuvant therapy. Cochrane Database Syst. Rev..

[14] Buffart L.M., Kalter J., Sweegers M.G., Courneya K.S., Newton R.U., Aaronson N.K., Jacobsen P.B., May A.M., Galvao D.A., Chinapaw M.J. (2017). Effects and moderators of exercise on quality of life and physical function in patients with cancer: An individual patient data meta-analysis of 34 RCTs. Cancer Treat. Rev..

[15] Kauffeldt K.D., Sabiston C.M., Santa Mina D., Tomasone J.R. (2021). An organizational approach to exploring the determinants of community-based exercise program implementation for breast cancer survivors. Support. Care Cancer.

[16] Kennedy M.A., Bayes S., Newton R.U., Zissiadis Y., Spry N.A., Taaffe D.R., Hart N.H., Galvão D.A. (2021). Implementation barriers to integrating exercise as medicine in oncology: An ecological scoping review. J. Cancer Surviv..

[17] Neugut A.I., Hillyer G.C., Kushi L.H., Lamerato L., Buono D.L., Nathanson S.D., Bovbjerg D.H., Mandelblatt J.S., Tsai W.-Y., Jacobson J.S. (2016). A prospective cohort study of early discontinuation of adjuvant chemotherapy in women with breast cancer: The breast cancer quality of care study (BQUAL). Breast Cancer Res. Treat..

[18] Denieffe S., Gooney M. (2011). A meta-synthesis of women’s symptoms experience and breast cancer: Meta-synthesis and breast cancer symptoms. Eur. J. Cancer Care.

[19] Peairs K.S., Barone B.B., Snyder C.F., Yeh H.-C., Stein K.B., Derr R.L., Brancati F.L., Wolff A.C. (2011). Diabetes Mellitus and Breast Cancer Outcomes: A Systematic Review and Meta-Analysis. J. Clin. Oncol..

[20] McCrary J.M., Goldstein D., Sandler C.X., Barry B.K., Marthick M., Timmins H.C., Li T., Horvath L., Grimison P., Park S.B. (2019). Exercise-based rehabilitation for cancer survivors with chemotherapy-induced peripheral neuropathy. Support. Care Cancer.

[21] Kleckner I.R., Kamen C., Gewandter J.S., Mohile N.A., Heckler C.E., Culakova E., Fung C., Janelsins M.C., Asare M., Lin P.-J. (2018). Effects of exercise during chemotherapy on chemotherapy-induced peripheral neuropathy: A multicenter, randomized controlled trial. Support. Care Cancer.

[22] Hasenoehrl T., Palma S., Ramazanova D., Kölbl H., Dorner T.E., Keilani M., Crevenna R. (2020). Resistance exercise and breast cancer–related lymphedema—a systematic review update and meta-analysis. Support. Care Cancer.

[23] Lane K., Worsley D., McKenzie D. (2005). Exercise and the Lymphatic System: Implications for Breast-Cancer Survivors. Sports Med..

[24] Singh B., Disipio T., Peake J., Hayes S.C. (2016). Systematic Review and Meta-Analysis of the Effects of Exercise for Those With Cancer-Related Lymphedema. Arch. Phys. Med. Rehabil..

[25] Kannan P., Lam H.Y., Ma T.K., Lo C.N., Mui T.Y., Tang W.Y. (2021). Efficacy of physical therapy interventions on quality of life and upper quadrant pain severity in women with post-mastectomy pain syndrome: A systematic review and meta-analysis. Qual. Life Res..

[26] Sozen T., Ozisik L., Calik Basaran N. (2017). An overview and management of osteoporosis. Eur. J. Rheumatol..

[27] Eastell R., Adams J.E., Coleman R.E., Howell A., Hannon R.A., Cuzick J., Mackey J.R., Beckmann M.W., Clack G. (2008). Effect of Anastrozole on Bone Mineral Density: 5-Year Results From the Anastrozole, Tamoxifen, Alone or in Combination Trial 18233230. J. Clin. Oncol..

[28] Thomas G.A., Cartmel B., Harrigan M., Fiellin M., Capozza S., Zhou Y., Ercolano E., Gross C.P., Hershman D., Ligibel J. (2017). The effect of exercise on body composition and bone mineral density in breast cancer survivors taking aromatase inhibitors: 12-Month Body Composition Changes in HOPE. Obesity.

[29] Pulido C., Vendrell I., Ferreira A.R., Casimiro S., Mansinho A., Alho I., Costa L. (2017). Bone metastasis risk factors in breast cancer. Ecancermedicalscience.

[30] Michael Y., Hoffe S. (2019). Epidemiology, Clinical Presentation, and Diagnosis of Bone Metastasis in Adults.

[31] Campbell K.L., Cormie P., Weller S., Alibhai S.M.H., Bolam K.A., Campbell A., Cheville A.L., Dalzell M.-A., Hart N.H., Higano C.S. (2022). Exercise Recommendation for People With Bone Metastases: Expert Consensus for Health Care Providers and Exercise Professionals. JCO Oncol. Pract..

[32] Wilk M., Kepski J., Kepska J., Casselli S., Szmit S. (2020). Exercise interventions in metastatic cancer disease: A literature review and a brief discussion on current and future perspectives. BMJ Support. Palliat. Care.

[33] Capozzi L.C., Daun J.T., Ester M., Mosca S., Langelier D., Francis G.J., Chang E., Mina D.S., Fu J.B., Culos-Reed S.N. (2021). Physical Activity for Individuals Living with Advanced Cancer: Evidence and Recommendations. Semin. Oncol. Nurs..

[34] Plana J.C., Galderisi M., Barac A., Ewer M.S., Ky B., Scherrer-Crosbie M., Ganame J., Sebag I.A., Agler D.A., Badano L.P. (2014). Expert consensus for multimodality imaging evaluation of adult patients during and after cancer therapy: A report from the American Society of Echocardiography and the European Association of Cardiovascular Imaging. Eur. Heart J. Cardiovasc. Imaging.

[35] Scott J.M., Nilsen T.S., Gupta D., Jones L.W. (2018). Exercise Therapy and Cardiovascular Toxicity in Cancer. Circulation.

[36] Howden E.J., Bigaran A., Beaudry R., Fraser S., Selig S., Foulkes S., Antill Y., Nightingale S., Loi S., Haykowsky M.J. (2019). Exercise as a diagnostic and therapeutic tool for the prevention of cardiovascular dysfunction in breast cancer patients. Eur. J. Prev. Cardiol..

[37] Kirkham A.A., Shave R.E., Bland K.A., Bovard J.M., Eves N.D., Gelmon K.A., McKenzie D.C., Virani S.A., Stöhr E.J., Warburton D.E.R. (2017). Protective effects of acute exercise prior to doxorubicin on cardiac function of breast cancer patients: A proof-of-concept RCT. Int. J. Cardiol..

[38] Borg G.A. (1998). Borg’s Perceived Exertion and Pain Scale.

[39] Siegel R.L., Miller K.D., Fuchs H.E., Jemal A. (2021). Cancer Statistics, 2021. CA Cancer J. Clin..

[40] Freedman R.J., Aziz N., Albanes D., Hartman T., Danforth D., Hill S., Sebring N., Reynolds J.C., Yanovski J.A. (2004). Weight and Body Composition Changes during and after Adjuvant Chemotherapy in Women with Breast Cancer. J. Clin. Endocrinol. Metab..

[41] Crew K.D., Greenlee H., Capodice J., Raptis G., Brafman L., Fuentes D., Sierra A., Hershman D.L. (2007). Prevalence of Joint Symptoms in Postmenopausal Women Taking Aromatase Inhibitors for Early-Stage Breast Cancer. J. Clin. Oncol..

[42] Saad F., Adachi J.D., Brown J.P., Canning L.A., Gelmon K.A., Josse R.G., Pritchard K.I. (2008). Cancer Treatment–Induced Bone Loss in Breast and Prostate Cancer. J. Clin. Oncol..

[43] Shaikh H., Bradhurst P., Ma L.X., Tan S.Y.C., Egger S.J., Vardy J.L. (2020). Body weight management in overweight and obese breast cancer survivors. Cochrane Database Syst. Rev..

[44] Nyrop K.A., Deal A.M., Shachar S.S., Basch E., Reeve B.B., Choi S.K., Lee J.T., Wood W.A., Anders C.K., Carey L.A. (2019). Patient-Reported Toxicities During Chemotherapy Regimens in Current Clinical Practice for Early Breast Cancer. Oncologist.

[45] Hsueh E.-J., Loh E.-W., Lin J.J.-A., Tam K.-W. (2021). Effects of yoga on improving quality of life in patients with breast cancer: A meta-analysis of randomized controlled trials. Breast Cancer.

[46] Eyigor S., Karapolat H., Yesil H., Uslu R., Durmaz B. (2010). Effects of pilates exercises on functional capacity, flexibility, fatigue, depression and quality of life in female breast cancer patients: A randomized controlled study. Eur. J. Phys. Rehabil. Med..

[47] Bekhet A.H., Abdallah A.R., Ismail H.M., Genena D.M., Osman N.A., El Khatib A., Abbas R.L. (2019). Benefits of Aerobic Exercise for Breast Cancer Survivors: A Systematic Review of Randomized Controlled Trials. Asian Pac. J. Cancer Prev..

[48] Bower J.E., Ganz P.A., Desmond K.A., Rowland J.H., Meyerowitz B.E., Belin T.R. (2000). Fatigue in Breast Cancer Survivors: Occurrence, Correlates, and Impact on Quality of Life. J. Clin. Oncol..

[49] Abrahams H.J.G., Gielissen M.F.M., Schmits I.C., Verhagen C.A.H.H.V.M., Rovers M.M., Knoop H. (2016). Risk factors, prevalence, and course of severe fatigue after breast cancer treatment: A meta-analysis involving 12 327 breast cancer survivors. Ann. Oncol..

[50] Juvet L.K., Thune I., Elvsaas I.K.Ø., Fors E.A., Lundgren S., Bertheussen G., Leivseth G., Oldervoll L.M. (2017). The effect of exercise on fatigue and physical functioning in breast cancer patients during and after treatment and at 6 months follow-up: A meta-analysis. Breast.

[51] Meneses-Echávez J.F., González-Jiménez E., Ramírez-Vélez R. (2015). Effects of supervised exercise on cancer-related fatigue in breast cancer survivors: A systematic review and meta-analysis. BMC Cancer.

[52] Schmidt M.E., Wiskemann J., Armbrust P., Schneeweiss A., Ulrich C.M., Steindorf K. (2015). Effects of resistance exercise on fatigue and quality of life in breast cancer patients undergoing adjuvant chemotherapy: A randomized controlled trial: Effects of resistance exercise on fatigue. Int. J. Cancer.

[53] Leach H.J., Danyluk J.M., Nishimura K.C., Culos-Reed S.N. (2016). Benefits of 24 versus 12 weeks of exercise and wellness programming for women undergoing treatment for breast cancer. Support. Care Cancer.

[54] Capozzi L.C., Lau H., Reimer R.A., McNeely M., Giese-Davis J., Culos-Reed S.N. (2012). Exercise and nutrition for head and neck cancer patients: A patient oriented, clinic-supported randomized controlled trial. BMC Cancer.

[55] Capozzi L.C., Boldt K.R., Easaw J., Bultz B., Culos-Reed S.N. (2016). Evaluating a 12-week exercise program for brain cancer patients: Exercise for brain cancer. Psycho-Oncology.

[56] Culos-Reed N., Dew M., Zahavich A., Wilson K., Arnason T., Mackenzie M., Brissette C., Van Patten C., Santa Mina D. (2018). Development of a Community Wellness Program for Prostate Cancer Survivors. Transl. J. Am. Coll. Sports Med..

[57] Finne E., Glausch M., Exner A.K., Sauzet O., Stoelzel F., Seidel N. (2018). Behavior change techniques for increasing physical activity in cancer survivors: A systematic review and meta-analysis of randomized controlled trials. Cancer Manag. Res..

[58] Covington K.R., Marshall T., Campbell G., Williams G.R., Fu J.B., Kendig T.D., Howe N., Alfano C.M., Pergolotti M. (2021). Development of the Exercise in Cancer Evaluation and Decision Support (EXCEEDS) algorithm. Support. Care Cancer.

[59] Stout N.L., Brown J.C., Schwartz A.L., Marshall T.F., Campbell A.M., Nekhlyudov L., Zucker D.S., Basen-Engquist K.M., Campbell G., Meyerhardt J. (2020). An exercise oncology clinical pathway: Screening and referral for personalized interventions. Cancer.

[60] Mina D.S., Sabiston C.M., Au D., Fong A.J., Capozzi L.C., Langelier D., Chasen M., Chiarotto J., Tomasone J.R., Jones J.M. (2018). Connecting people with cancer to physical activity and exercise programs: A pathway to create accessibility and engagement. Curr. Oncol..

[61] Silver J.K., Baima J., Mayer R.S. (2013). Impairment-driven cancer rehabilitation: An essential component of quality care and survivorship: Impairment-Driven Cancer Rehabilitation. CA Cancer J. Clin..

[62] Guralnik J.M., Simonsick E.M., Ferrucci L., Glynn R.J., Berkman L.F., Blazer D.G., Scherr P.A., Wallace R.B. (1994). A Short Physical Performance Battery Assessing Lower Extremity Function: Association With Self-Reported Disability and Prediction of Mortality and Nursing Home Admission. J. Gerontol..

[63] Crooks V., Waller S., Smith T., Hahn T.J. (1991). The Use of the Karnofsky Performance Scale in Determining Outcomes and Risk in Geriatric Outpatients. J. Gerontol..

[64] Azam F., Latif M.F., Farooq A., Tirmazy S.H., AlShahrani S., Bashir S., Bukhari N. (2019). Performance Status Assessment by Using ECOG (Eastern Cooperative Oncology Group) Score for Cancer Patients by Oncology Healthcare Professionals. Case Rep. Oncol..

[65] Almirall D., Nahum-Shani I., Sherwood N.E., Murphy S.A. (2014). Introduction to SMART designs for the development of adaptive interventions: With application to weight loss research. Transl. Behav. Med..

[66] Buchholz S.W., Wilbur J., Halloway S., Schoeny M., Johnson T., Vispute S., Kitsiou S. (2020). Study protocol for a sequential multiple assignment randomized trial (SMART) to improve physical activity in employed women. Contemp. Clin. Trials.

[67] Pfammatter A.F., Nahum-Shani I., DeZelar M., Scanlan L., McFadden H.G., Siddique J., Hedeker D., Spring B. (2019). SMART: Study protocol for a sequential multiple assignment randomized controlled trial to optimize weight loss management. Contemp. Clin. Trials.

